# Biana: a software framework for compiling biological interactions and analyzing networks

**DOI:** 10.1186/1471-2105-11-56

**Published:** 2010-01-27

**Authors:** Javier Garcia-Garcia, Emre Guney, Ramon Aragues, Joan Planas-Iglesias, Baldo Oliva

**Affiliations:** 1 Structural Bioinformatics Lab. (GRIB). Universitat Pompeu Fabra-IMIM. Barcelona Research Park of Biomedicine (PRBB). 08003-Barcelona, Catalonia, Spain

## Abstract

**Background:**

The analysis and usage of biological data is hindered by the spread of information across multiple repositories and the difficulties posed by different nomenclature systems and storage formats. In particular, there is an important need for data unification in the study and use of protein-protein interactions. Without good integration strategies, it is difficult to analyze the whole set of available data and its properties.

**Results:**

We introduce BIANA (Biologic Interactions and Network Analysis), a tool for biological information integration and network management. BIANA is a Python framework designed to achieve two major goals: i) the integration of multiple sources of biological information, including biological entities and their relationships, and ii) the management of biological information as a network where entities are nodes and relationships are edges. Moreover, BIANA uses properties of proteins and genes to infer latent biomolecular relationships by transferring edges to entities sharing similar properties. BIANA is also provided as a plugin for Cytoscape, which allows users to visualize and interactively manage the data. A web interface to BIANA providing basic functionalities is also available. The software can be downloaded under GNU GPL license from http://sbi.imim.es/web/BIANA.php.

**Conclusions:**

BIANA's approach to data unification solves many of the nomenclature issues common to systems dealing with biological data. BIANA can easily be extended to handle new specific data repositories and new specific data types. The unification protocol allows BIANA to be a flexible tool suitable for different user requirements: non-expert users can use a suggested unification protocol while expert users can define their own specific unification rules.

## Background

Advances over the past years have yielded a vast amount of experimental high-throughput data on relationships between biological entities such as proteins and genes [1-3]. This information is spread across multiple databases, containing different types of the stored data, accession nomenclature and interface (HPRD [4], MINT [5], BioGrid [6], IntAct [7], MIPS [8]). The main difficulty to merge the data provided in these databases is having distinct identifiers for the same biological entity [9]. Therefore a protocol that unifies biological data independently of the identifiers used on each data source is required. A number of works have addressed the standardization of nomenclature and format of biological entities (HGNC [10], HUPO-PSI [11]) while some others have tackled the problems of redundant data produced by cross-references (IPI [12], PIANA[13], BNDB[14], APID [15], UniHI [16], bioDBnet [17], ONDEX [18] and iRefIndex [19]).

Here we present BIANA, a tool for biological database unification and network management that can be used as a standalone application or as a plugin for Cytoscape. BIANA uses a generic method to find entries of a given molecule that are equivalent across different biological data repositories. Moreover, BIANA incorporates and empowers a variety of network analysis methods through NetworkX [20]. In addition to unifying all major biological repositories, BIANA is easily adaptable to newly created data repositories. BIANA is an extension of the Protein Interaction and Network Analysis (PIANA) [13], which was focused on protein-protein interactions. BIANA bridges the network visualization of Cytoscape and the network analysis capabilities of NetworkX and Cytoscape with customizable data unification for relationships between genes and their products. BIANA addresses the challenge of unambiguously gathering available data for biological entities of interest and working with networks built with its relationships. BIANA network-analysis capabilities have been compared with other programs under the same set of features as those presented in Cline et al. [21] (see Table 1). BIANA data integration capabilities have also been compared with up-to-date software focused on data integration (see Table 2).

**Table 1:** Comparison of network analysis platforms.

Feature	CY	GM	VA	OS	CD	AR	IN	GG	PI	PR	BL	PA	BI
Free for academic use	X	X	X	X	X				X	X	X	X	**X**

Free for commercial use	X	X	X		X				X	X	X		**X**

Open source	X	X							X	X	X		**X**

Curated pathway/network content		X		X		X	X	X					

Standard file format support	X		X		X				X	X		X	**X**

User-defined networks/pathways	X	X	X	X	X	X	X	X	X	X	X	X	**X**

Functionality to infer new pathways	X		X			X		X	X				**X**

GO/pathway enrichment analysis	X	X	X				X	X					**X**

Automated graph layout	X		X	X	X	X	X	X		X	X	X	**X**

Complex criteria for visual properties		X				X	X	X		X	X	X	

Multiple visual styles	X		X	X		X	X			X			**X**

Advanced node selection	X		X	X		X	X	X	X	X	X	X	**X**

Customizable gene/protein database		X	X			X		X	X				**X**

Rich graphical annotation		X	X				X	X				X	**X**

Statistical network analysis	X		X				X	X	X		X		**X**

Extensible functionality: plugins or API	X		X		X	X	X	X	X				**X**

Quantitative pathway simulation					X	X							

BIANA has been compared with the same programs and using the same set of features as the ones presented in [21]. Compared software: CY, Cytoscape [55]; GM, GenMAPP [58]; VA, VisANT [59]; OS, Osprey [60]; CD, CellDesigner [61]; AR, Ariadne Genomics Pathway Studio [62]; IN, Ingenuity Pathways Analysis http://www.ingenuity.com; GG, GeneGO http://www.genego.com; PI, PIANA [13]; PR, ProViz [63]; BL, BioLayout [64]; PA, PATIKA [65]; BI, BIANA.

**Table 2:** Comparison of biological information integration softwares.

	Feature	BI	PI	AP	AP2	BN	UH	MI	ON	iRI
**Data types**	Supports multiple biomolecule types (protein, gene, compound...)	X				X		X	X	
	
	Supports multiple relation types (interaction, complex, pathway...)	X				X	X	X	X	
	
	Supports multiple data descriptor/identifiers types	X	X	X	X	X	X		X	X
	
	***User extensible to new user defined data types and attributes***	X								

**Data Unification**	***User specific data unification***	X							(1)	
	
	***Standard user can extend to new data repositories***	X							(1)	

**User Interface**	Standalone Graphical Interface					X			X	
	
	Scripting/Command line	X	X			X			X	
	
	Provides a webserver	X		X		X	X	X		X
	
	Provides a plugin for Cytoscape	X			X			X		X

**Network analysis**	Adds network analysis methods	X	X			X			X	

**Availability**	Open Source	X	X			X			X	

**Installation**	Does not require additional software			X	X		X	X	X	X
	
	Standalone application (runs locally)	X	X			X			X	

BIANA has been compared with other biological databases integration software/webservers. Compared software: PI, PIANA [13]; AP, APID [15]; AP2, APID2NET [66]; BN, BNDB [14]; UH, UniHI [67]; MI, MIMI [68], ON, ONDEX [18], iRI, iRefIndex [19]. (1)According to the original manuscript, "The installation and use of the data integration methods is still command line driven and requires technical expertise to install, configure and use this component of the ONDEX system".

## Implementation

### Software architecture

**Figure 1:**
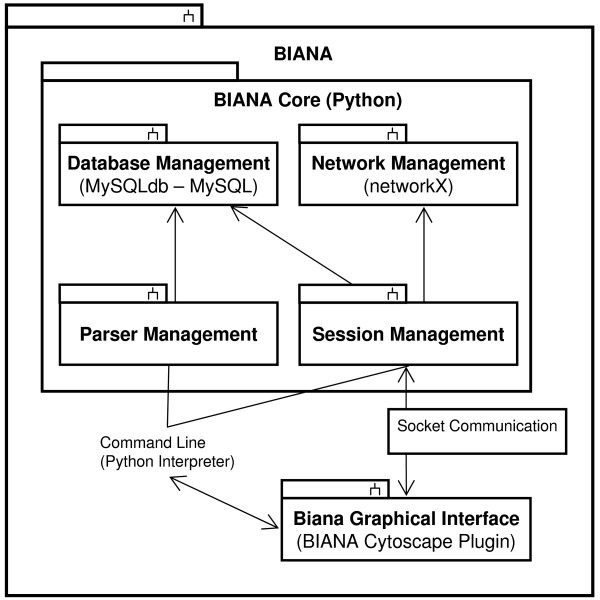
**BIANA Architecture**. BIANA is composed of 4 different modules: Database Module, Parser Module, Network Module and Session Management Module. Database Module handles communication between BIANA and MySQL database. Parser Module imports data into BIANA database. Network Module performs all network procedures using NetworkX package. Session Management Module to handle biological data sets and networks. BIANA Cytoscape Plugin is a separate interface that communicates Cytoscape with BIANA through a socket. BIANA framework can be executed with Python interpreter (as well as command line python scripts) or in Cytoscape with a plugin.

BIANA is a Python framework composed of four different modules (Figure 1): 1) Database Management (handles communication between BIANA and MySQL database); 2) Parser Management (imports data into BIANA database); 3) Network management (performs networking operations using NetworkX package); and 4) Session Management (to manage biological data sets and their networks). The Cytoscape Plugin is a separate and user friendly interface to BIANA (the plugin communicates with BIANA & Cytoscape through a socket).

### Data model

**Figure 2:**
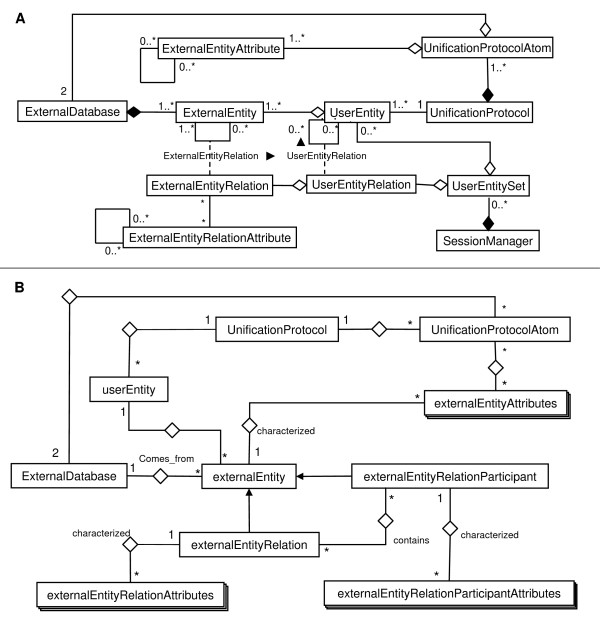
**BIANA Data Model**. **A) BIANA Data Model Diagram**. Schematic UML (Unified Modeling Language) representation of the data entries and their relationships in BIANA. Explanation of each element is given in the text. **B) BIANA Database Architecture**.

BIANA uses a high level abstraction schema (see Figure 2) to handle databases providing biological information (i.e. individual entries and their relationships). Any data source that contains biologic or chemical data parsed by BIANA is defined as an *external database*. Similarly, BIANA adopts the concept of *external entity*, corresponding to entries in external databases, and integrates these *external entities *coming from different *external databases*. For example, a Uniprot entry (a protein), a GenBank entry (a gene), an IntAct interaction (a protein-protein physical interaction), a KEGG pathway (a metabolic relationship) or a PFAM alignment are all represented as external entities. In order to achieve data uniformity, the participants of a partnership and its relationship are considered *external entities*, whereas the relation itself is annotated as *external entity relation *which is a subtype of external entity. *External entities *are characterized by several *attributes*, such as database identifiers, sequence, taxonomy, description or function. Each *external entity relation *is further characterized by some attributes (i.e. detection method or reliability). Alternatively, the participants in *external entity relations *can have their particular *attributes *(i.e. role or cardinality). BIANA unifies external data inserted into its database based on a specific protocol. This protocol, called *unification protocol*, consists of a set of rules (*unification protocol atom*) that determine how data in various data sources are combined (crossed). Each rule is composed of attributes crossed and the pair of *external databases *used. Two *external entities *(each coming from one of these external databases) will be considered "equivalent" provided that they share the same annotation (value) for the specified attribute(s) in the rule. The set of *external entities *that are decided to be equivalent with respect to a given unification protocol is called *user entity *(group of biomolecules that are considered equivalent). *User entities *inherit all the attributes of their included external entries. Each *external entity *can belong to a single *user entity*, unless the database is defined as *promiscuous database*, where a single external entity can belong to multiple user entities.

### BIANA User Interface

Depending on user background and objectives, BIANA offers three user interfaces: 1) *command line interface *for most advanced users (usually bioinformaticians), interested in using all BIANA functionalities, network analysis procedures provided by *NetworkX *and other *Python *modules, and interested in automatic processes by using scripts; 2) *Cytoscape Plugin interface *benefits users interested in the interactivity offered by a graphical interface without lost of functionality. The plugin has one main advantage: it provides a command line terminal to help most advanced users to create scripts that run in BIANA as a command line application or to execute other *Python *or *NetworkX *commands; and 3) *online interface *for non-expert users who prefer using a web-server that provides only basic functionalities of BIANA. This is the easiest access and it does not require installation. The web-server uses a pre-stored database of interactions with a default unification protocol, but prevents benefiting from the user-driven unification capabilities.

## Results and Discussion

**Figure 3:**
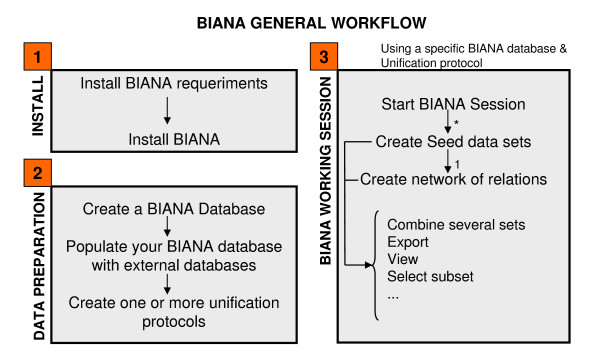
**BIANA Workflow**. BIANA working procedure involves at least 3 steps: 1) Install BIANA package and Cytoscape plugin if required; 2) Populate BIANA database and create unification protocols; and 3) Start a working session.

BIANA working procedure has two preliminary steps (Figure 3): 1) installing BIANA package and Cytoscape plugin; and 2) populating a BIANA-database store plus creating one or more unification protocols for this database. After these steps, a working session can be started.

### BIANA database creation

**Figure 4:**
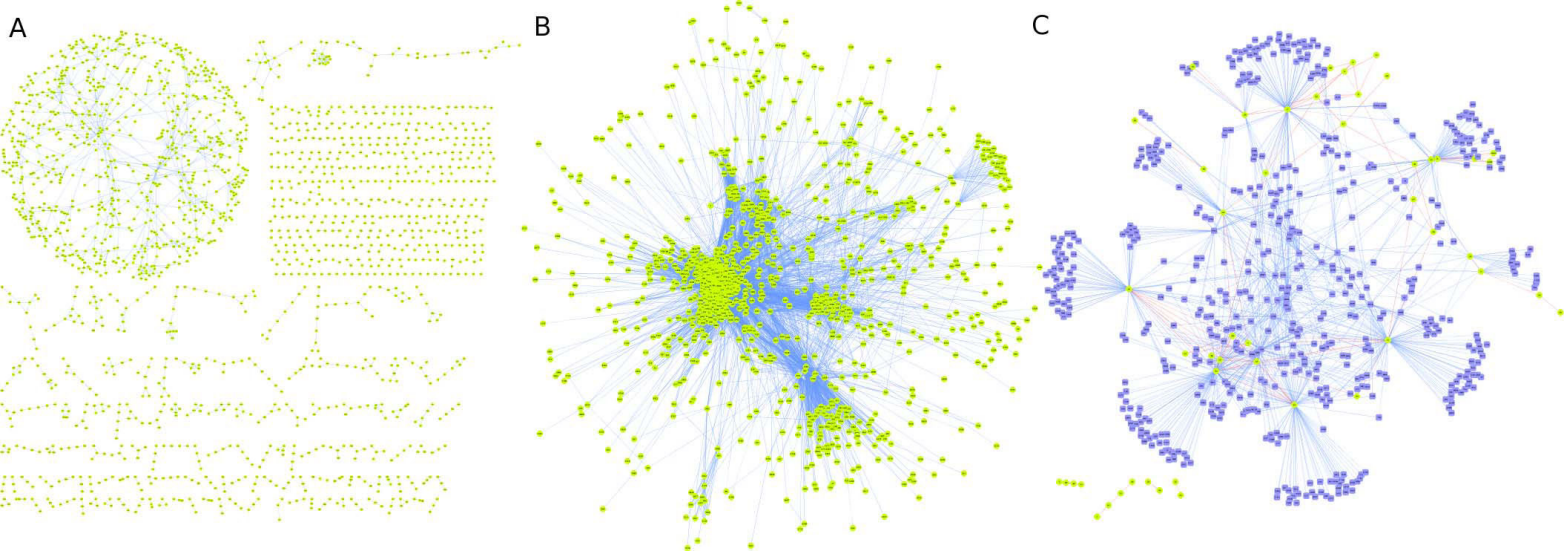
**BIANA networks created from user provided datasets**. BIANA Cytoscape plugin has been used to generate the relation networks of three different user specific datasets (data is available at http://sbi.imim.es/web/BIANA.php). Represented entities are: *Proteins *(green nodes), *genes *(blue nodes), *interactions *and *metabolic relations *(blue edges) and *cooperation *(red edges). **A) **Metabolic network reconstruction where a relation is established and scored between each pair of possible chained enzymatic reactions. A chained reaction between *enzyme A *and *enzyme B *is possible when there is at least one chemical compound in the intersection, acting at the same time as product of *enzyme A *and substrate of *enzyme B*. The network has been filtered with score greater than 1.2 (score is based on the plausibility of observing chemical compounds in the intersection, according to their own frequency and the frequency of other products of enzyme A and other substrates of enzyme B that do not take part in chaining reactions). **B) **Protein-protein interaction network predicted from sequences/structure distant patterns as described in Espadaler et al. [22]. Only human proteins are shown and interactions coming from set I3 (see a detailed explanation in the original work). **C) **Network representation of cooperative transcription factors and their regulated genes described at Aguilar et al. [23]. Only transcription factors cooperating with others have been represented.

After BIANA installation, the next step consists of creating and populating a BIANA database with desired *external databases*. BIANA offers several parsers for most well known biological databases (Table 3) and includes most common attributes (identifiers as *UniprotAccession*, *UniprotEntry *and *GeneID*, descriptive attributes as *description *and *function *among others). BIANA also offers the possibility to add new parsers for other third-party databases or to add user provided data using a generic format, as well as to add new attributes (i.e. new identifier types or new descriptive types). New parsers for other databases or for user provided data can be uploaded to our project website http://sbi.imim.es/web/BIANA.php?page=biana.parsers and be shared with the rest of BIANA users. For example, we provide three different datasets used in our group (see datasets at http://sbi.imim.es/web/BIANA.php: 1) one dataset contains EC codes (as nodes) and the relationships between them defined by the compounds involved in their reactions (as edges), and it includes several new features such as the number of common metabolites and the direction of the reaction (Figure 4A); 2) a second set contains interactions predicted from sequences/structure distant patterns [22] (Figure 4B); and 3) we have also included a set of transcription factors and their regulated genes plus information on their cooperativity [23] (Figure 4C).

**Table 3:** Default *external database *parsers provided by BIANA.

External Database (checked version)	Details
**General databases (Sequence, identifiers, and cross-reference databases)**	

Uniprot [31] (Release 14.1)	Protein sequence, identifiers and functional information (domain composition, description, function...). Both Swiss-prot (manually curated) and TrEMBL (automatically annotated) can be inserted into BIANA. **Protein sequences **and **multiple attributes **are inserted into BIANA.

GenPept from GenBank [69] (FASTA formatted file) (Release 167)	Protein sequences translated from the GenBank database. GenBank is the NIH genetic sequence database, a collection of all publicly available DNA. **Protein sequences **and **identifiers **are inserted into BIANA.

Non-redundant Blast Database (FASTA formatted file) (August 2008)	BLAST Non-redundant database from NCBI. Non-redundant protein sequence database with entries from GenPept, SwissProt, PIR, PDF, PDB and NCBI RefSeq. **Protein sequences **and **identifiers **are inserted into BIANA.

International Protein Index (IPI) [12] (September 2008)	Integrated database for proteomics experiments. **Protein sequences **and **identifiers **for Human, Mouse, Rat, Zebrafish, Arabidosis, Chicken and Cow are inserted into BIANA.

HUGO Gene Nomenclature Committee (HGNC) (September 2008)	Approved unique gene symbols for each human gene. **Cross-references **are inserted into BIANA. http://www.genenames.org

Cluster of Orthologous Genes (COGs) [70] (2003)	Collection of orthologous protein sets for prokaryotes and eukaryotes. ***Protein identifiers ***and ***COG ***groups are inserted into BIANA.

**Ontologies**	

Gene Ontology (GO) [33] (version 1.2)	The Gene Ontology provides a controlled vocabulary to describe gene and gene product attributes in any organism. It allows to link in BIANA between *GO ID *and ***GO name ***and ***type***.

PSI-MI obo	Controlled vocabulary and ontology for molecular interactions and their detection methods. Provides the information about and the relation between **method ID **and **method name**. http://psidev.info/MI

NCBI Taxonomy [71]	The NCBI taxonomy database contains the names of all organisms that are represented in the genetic databases. It allows to link between ***taxonomy ID ***identifier to ***Taxonomy name ***attribute.

Structural Classification of Proteins (SCOP) [34]	Manually curated database with a comprehensive description of the structural and evolutionary relationships between all proteins whose structure is known. It has a hierarchical classification of the structural domains.

**Relation databases**	

PSI-MI 2.5 Format [11]	Data exchange format for molecular interactions. The following protein-protein interaction databases can be inserted into BIANA: IntAct [7] (September 2008), DIP [72] (2008.07.08), HPRD [36] (Release 7), BioGrid [6] (v2.0.44), MPACT [73] (April 2007), MINT [5] (2008.05.21)

Biopax Level 2 Format	Data exchange format for biological pathway data. The following databases can be inserted into BIANA: Reactome [35] (September 2008)

iRefIndex [19]	A consolidated protein interaction database with provenance. (April 2009)

Kyoto Encyclopedia of Genes and Genomes (KEGG) [32]	Kegg Ligand (chemical compounds, drugs, glycans and reactions), Kegg genes (genomes, genes and proteins) and Kegg orthology (ortholog annotation) are inserted into BIANA.

STRING [74]	Database of known and predicted protein interactions. Includes direct (physical) and indirect (functional) associations.

BIANA provides the following parsers for common public biologic databases. Updated database parsers can be uploaded in the project webpage http://sbi.imim.es/web/BIANA.php.

Furthermore, we created a BIANA-database for the convenience of users as an initial starting point to start using BIANA (either from command line or from Cytoscape) whose accessibility information is given on the project web page. The online database contains information parsed from the following resources (note that BIANA web-server uses a more complete database whose details can be found on the project page as well): Uniprot Swissprot, Gene Ontology (GO), NCBI Taxonomy database, IntAct database, MINT database, PSI-MI ontology, Reactome, plus specific tutorial sets for the theoretical example and SBI datasets used in previous works [22,23].

### Unification protocols

**Figure 5:**
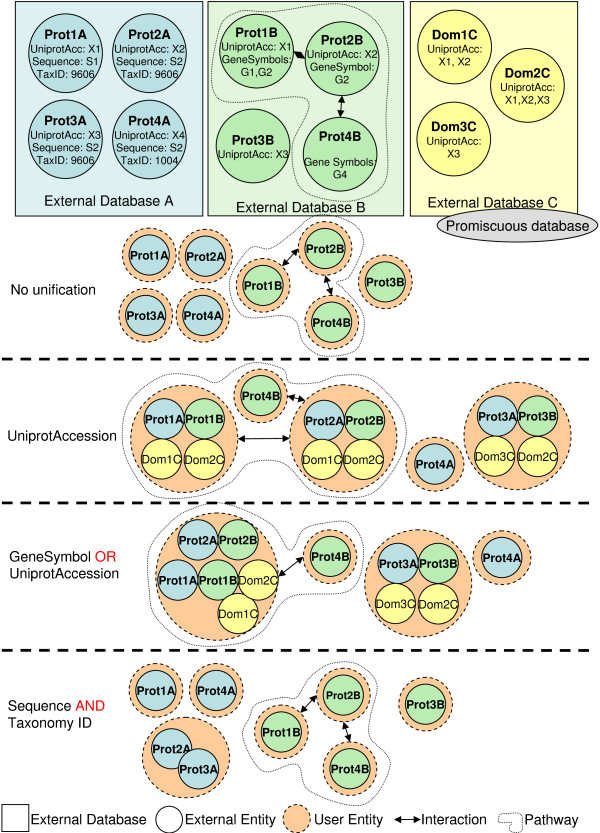
**BIANA Unification**. Example where three different *unification protocols *are applied to three *external databases *(each *external database *is represented with a different color). BIANA network nodes are individual *user entities*. Each *user entity *consists of a set of equivalent *external entities*. Each *external entity *can belong to a single *user entity*, unless the database is defined as *promiscuous database*, where a single external entity can belong to multiple user entities. *External entities *in *promiscuous databases *can not form a *user entity *by themselves. In this theoretical example, in order to show the importance of the *unification protocol*, it can be observed *Prot1A *is merged with *Prot1B *when unifying by *UniprotAccession *identifier, while they are not merged if unification is done by *Sequence *and *taxonomyID*. However, when unified by *UniprotAccession *or *geneSymbol*, *Prot1A*, *Prot1B*, *Prot2A *and *Prot2B *are merged.

Once a database has been loaded in BIANA MySQL server, next step consists on the integration of data and its relationships. BIANA utilizes *user entries *defined by a certain *unification protocol *chosen by the user. A recommended *unification protocol *is also provided for non-expert users (Table 4), but users are free to create their own unification protocols according to their needs. As an example, a user may be interested in creating a unification protocol defined by crossing entities using sequence and taxonomy information between two or more databases (similarly to the integration protocol used in PIANA [13] and the Redundant Object Group used in *iRefIndex *[19]). In another example, the user can use sequence and taxonomy as well as Uniprot accession code between two or more databases. Different *unification protocols *applied to the same external databases can lead to different outcomes (see Figure 5). The advantages of this unification approach are: 1) BIANA database only contains raw data (with exactly the same nomenclature and identifiers of the original data source), therefore it does not entail any assumption on data integration and it allows the user to specify how the integration should be done. 2) Information from a single database or the combination of multiple databases can be selected by the user in each experiment. And 3) the original data can be easily tracked back with all its user interfaces (API, BIANA Cytoscape Plugin and WebServer).

**Table 4:** Recommended unification protocols.

External Databases	Attributes (identifiers)
Uniprot, GeneBank, IPI, KeggGene, COG, String	ProteinSequence AND taxID

Uniprot, HGNC, HPRD, DIP, MPACT, Reactome, IPI, BioGrid, MINT, IntAct, String	UniprotAccession

Uniprot, String	UniprotEntry

Uniprot, HGNC, HPRD, DIP, String	GeneID

Uniprot, SCOP(promiscuous)	PDB

List of *external databases *and the attributes (identifiers) proposed to be used in a unification protocol.

The data available on the online BIANA database is unified with respect to the following criteria of equivalence: All entries coming from any biological data repository are grouped in the same user entity if and only if they share *UniprotAccession *code "or", both *sequence *and *Taxonomy *identifier, "or" *GeneID*. In particular, when BIANA is queried for *ERF1_YEAST *as Uniprot Entry code, it groups all the entries coming from Swissprot (*P12385*), IntAct (*EBI-6533*), MINT (*MINT-560710*) and Reactome (*REACT_1034*) in one user entity (a node in BIANA; since they have been annotated with the same Uniprot Accession or sequence and taxonomy or GeneID to that of *ERF1_YEAST*).

### BIANA working-session capabilities

After creating a BIANA database and creating one or more *unification protocols*, a BIANA working session includes the following capabilities:

#### -Network creation

Integrated biological entities consisting of proteins, genes or drugs are considered as nodes of a network, while relationships between them such as interactions, gene regulation, metabolic reactions or signal transduction are edges. The first step to obtain a network is the acquisition of an initial set of seed nodes (i.e. the biological entities of interest). Then, BIANA creates the network of relationships by retrieving their direct partners (nodes interacting with the seeds). The network construction procedure can run iteratively, defining successive levels of partnership (seed nodes are in level 0, partners of the seed nodes define the level 1 and nodes connected with nodes in level *i *define the level *i+1*). Users can create the network using various types of relationships and impose restrictions based on the attributes of these relationships or their nodes (for example, by restricting on the detection method). Networks are widely employed to study specific pathologies [24].

#### -Analysis of networks

BIANA grants access to most of existing methods for the analysis of networks through *NetworkX *and *Cytoscape*: finding shortest paths and connected components, calculating node degrees and network connectivity, etc. In addition, BIANA includes new methods such as network randomization, node and edge tagging, calculation of linker degree based on node tags [25], intersection and merging of networks. Recently, BIANA has been used in simplifying the improvement of fold recognition using protein-protein interactions [26] or in modeling and analysis of aneurism-related molecular interactions using text-mining seed-nodes [27].

#### -Predictions of edges

BIANA predicts novel relationships by transferring existing edges between nodes with common properties. Basically, let *x, y, z *be biological entities obtained with the unification approach. An interaction is predicted between *x *and *y *if: i) *x *is observed to interact with *z*; and ii) *y *shares some attributes (decided by the user, i.e. PFAM domains, SCOP domains, or sequence similarity using cut-offs based on e-value or percentage of identity) with node *z*. This is an extension of the definition of interologs [28] using other relationships different than orthology. For example, we generated protein-protein interaction networks from proteins we compared them with networks of protein-protein interaction predictions based on the transfer of interactions between proteins (i.e. y and z) whose 90% of its sequence could be aligned with at least 90% of sequence similarity (Table 5).

**Table 5:** Comparison of three different networks at level 1.

Disease	Keywords	Initial Set	PPI	PPI + inferred interactions
Cancer	Cancer, tumor, metastasis	985 (93)	2782 (251)	6272 (489)

Diabetes	Diabetes	86 (10)	284 (19)	2121 (54)

Alzheimer	Alzheimer	30 (4)	138 (6)	1098 (12)

Comparison of three different networks at level 1 using reported protein-protein interactions vs. using inferred interactions by sequence homology. A BIANA database has been created using the following databases: Uniprot Swissprot, IntAct, MINT, BioGrid, DIP and HPRD. Three different initial data sets related with three different pathologies have been created by a keyword search in fields *Disease*, *Keyword*, *Description *and *Function*. Two networks at level 1 have been created for each set: 1) using reported protein-protein interactions by third-party databases and 2) using inferred interactions by using sequence similarity (see text for details). For each network we calculated the number of proteins involved in the pathologies according to HEFalMp [75] with a p < 0.00001 (shown in parenthesis). By using inferred interactions a higher number of candidates are retrieved.

#### -Unification backtracking

As BIANA database architecture and access is pretended to be transparent for users, BIANA offers the possibility of backtrack the results of the unification protocol with the information as defined in original sources. For example, in the BIANA Cytoscape Plugin, users can check all entries from external databases fused into a single node during the unification protocol. Users can also check which are the exact relationships defined by external databases. These options are explained in the first example of the tutorial. In summary, when selecting the option "*View set details*" the user can select one or more nodes in the table and click the button "*View details*". This option shows in a new table all the original entries fused for each node (for example, a BIANA user entity node can contain an entry from the Uniprot Database, an entry from IPI database, some nodes from a protein-protein interaction database, etc). A similar procedure can be applied to show the relationships as defined in the original sources.

### Example: Investigating relationships between pathologies using BIANA

We have used the study case of the relationship between the networks of genes involved in the pathologies of Alzheimer and diabetes diseases. Under the context of systems and network biology, researchers are interested in discovering actors involved in diseases, their relationships and key shared elements on the organism level. Considering that Alzheimer's disease (AD) and diabetes are shown to be coupled, where having diabetes bears an increased risk for AD [see [29] and [30] for reviews], as an example of the use of BIANA, we look for proteins playing a role both in Alzheimer's disease and diabetes. BIANA is perfectly suited for pursuing such kind of tasks where one needs to fetch species-wide sequence annotation and interaction information spread across various data resources. For this example we integrated data from publicly available proteome knowledge bases such as Uniprot [31], Kegg [32], IPI [12], GO [33], SCOP* [34], HGNC [10] and major interaction data resources such as Reactome* [35], IntAct* [7], HPRD* [36], Biogrid* [6], MINT* [5]. All the databases with a star (*) are inserted as promiscuous. All of the listed databases are unified based on Uniprot Accession identifier; sequence in combination with Taxonomy identifier; and GeneId identifier. In addition to these unification rules, the data coming from SCOP & Uniprot databases are further unified using PDB codes. For step-by-step details of this case study we suggest to follow the chapter 6 of the tutorial and a video summary.

Using BIANA we identified proteins interacting with an Alzheimer or diabetes associated protein in the mouse proteome (since mouse is one of the most frequently used model organism in studies focused on AD and diabetes). Among the proteins contained in the intersection of the protein-protein interaction network, the Mitogen-activated protein kinase (MAPK8; aka JNK1) interacts both with proteins associated with AD and diabetes (in particular, the products of APP and MAPK8IP1 genes, respectively). Strikingly, JNK1 has been demonstrated to be involved in maintenance of neuronal mictrotubules [37], in beta-amyloid-mediated stabilization of p53 [38], and in cell death in the brain of patients with AD [39], but also in major risk factors of diabetes type II such as insulin resistance [40,41] and adiposity [42].

In order to extend our knowledge, we created interaction networks for all proteins stored in BIANA-database that were associated with AD and diabetes with independence of the proteome specie (thus, being not restricted to mouse). This was possible thanks to the unification protocol used in BIANA. Next, we filtered the proteins contained in these two networks, so that only proteins linked at least to two Alzheimer-associated proteins or to two diabetes-associated proteins were taken into account. Then, we extracted the intersection of these two subsets with BIANA and we selected those proteins that had not been attributed to any of the pathologies in the initial sets. Remarkably, we found direct supporting evidence in the literature that some of the proteins in this selected set were products of genes that had been reported to play key roles in both AD and diabetes (for example, CamKII is related with AD [43-45] and diabetes [46,47]; and GSK3b is related with AD [48,49] and diabetes [50,51]).

Finally, we included predicted interactions (i.e. we transferred interactions from proteins to their homologs pairs). We defined interaction predictions based on the transfer of interactions between proteins with at least 90% of sequence similarity and 90% of sequence covered by the alignment. This increased the initial list of potential mediators from 51 to 221 (see more details in tutorial example). A visual inspection on the list yielded some new interesting candidates to be evaluated, such as *calreticulin *or *drebrin*. For example, chaperone *calreticulin *appeared in the literature related with AD [52] and also with insulin receptors [53]. We also found *drebrin*, which has been related with AD and diabetes in the literature [54]. Clearly, this example was easily done thanks to the protocols of unification and network handling capabilities of BIANA.

## Conclusions

We have presented BIANA, a software framework designed to integrate several sources of biological data, exploit its relationships and facilitate its analysis. BIANA introduces an abstract data model to allow user-defined biological database unification and an easy to use interface for network creation and analysis. In order to make sure that BIANA would be freely accessible by anybody, BIANA framework uses either free open-source software or publicly available free software. For users who want to skip the software requirements, we provide BIANA web server at the price of loosing freedom on how to decide data unification, relinquishing to incorporate user-defined data and obliging simplified network analysis and visualization protocols.

The main advantage of BIANA against existing software is its design, which allows adding user specific data types and allows the user to handle his own unification protocol. However, unification is a non-trivial problem for non-expert users in bioinformatics. Therefore, we recommend a unification protocol for the databases for which we have provided a parser in our web page. BIANA website also offers a repository where users can download/upload new parsers for other third-party databases and make their own parsers available for the scientific community. Additionally, BIANA helps to handle the network, to expand with predictions or combined resources of information and to extract biologically relevant information from the network (as it was shown in the case study proposed in the example of AD and diabetes).

We believe BIANA will be of high interest for users who want to incorporate their own data on the analysis with other available biological data sources. It is also noteworthy that other software (or repositories) integrating several sources of interactions can only distribute data if there are no restrictions (or copyright agreements are fulfilled), while in BIANA the user is free to download interactions from official sites and freely integrate them. The capability of executing BIANA as a Cytoscape plugin allows users to benefit from existing Cytoscape plugins in a complementary manner; and the capability of executing BIANA through its Python API allows users to write scripts to access and analyze their data automatically.

## Availability and requirements

• **Project name: **BIANA. Biologic Interactions and Network Analysis.

• **Project home page: **http://sbi.imim.es/web/BIANA.php

• **Operating systems: **UNIX based systems, Windows

• **Programming language: **Python (BIANA), JAVA (Cytoscape plugin)

• **Other requirements: **In Windows all requirements are embedded in the software installer. In UNIX based systems it requires g++ compiler, Python2.5 and MySQL server 2.52. BIANA Cytoscape Plugin requires Cytoscape 2.6.0 [55]. In case one is interested in relations of biological data derived from sequence similarity (such as networks based on interology), CD-HIT [56] and BLAST [57] are also required.

• **License: **GNU GPL (GNU General Public License)

• **Restrictions: **Not applied

## Authors' contributions

BO conceived of the BIANA project and provided scientific guidance. BIANA software architecture was mainly designed by JGG with contributions of EG. JGG and EG implemented the code for the framework and the plugin and performed analyses. RA implemented the web server whose user interface is later improved by JGG. JP did the analysis on the Reactome network. JGG, EG wrote the manuscript and JP, RA and BO contributed to the final version. All authors read and approved the final manuscript.
